# Case Study of Polyvinylidene Fluoride Doping by Carbon Nanotubes

**DOI:** 10.3390/ma14061428

**Published:** 2021-03-15

**Authors:** Pavel Kaspar, Dinara Sobola, Klára Částková, Rashid Dallaev, Eva Šťastná, Petr Sedlák, Alexandr Knápek, Tomáš Trčka, Vladimír Holcman

**Affiliations:** 1 Department of Physics, Faculty of Electrical Engineering and Communication, Brno University of Technology, Technická 2848/8, 616 00 Brno, Czech Republic; sobola@feec.vutbr.cz (D.S.); xdalla03@stud.feec.vutbr.cz (R.D.); sedlakp@feec.vutbr.cz (P.S.); trcka@feec.vutbr.cz (T.T.); holcman@feec.vutbr.cz (V.H.); 2 Central European Institute of Technology BUT, Purkyňova 123, 612 00 Brno, Czech Republic; klara.castkova@ceitec.vutbr.cz (K.Č.); eva.jindrova@ceitec.vutbr.cz (E.Š.); 3 Department of Inorganic Chemistry and Chemical Ecology, Dagestan State University, Makhachkala, St. M. Gadjieva 43-a, 367015 Makhachkala, Russia; 4 Department of Ceramics and Polymers, Faculty of Mechanical Engineering, Brno University of Technology, Technická 2, 616 69 Brno, Czech Republic; 5 Institute of Scientific Instruments of the Czech Academy of Sciences, Královopolská 147, 612 64 Brno, Czech Republic; knapek@isibrno.cz

**Keywords:** polyvinylidene fluoride, carbon nanotubes, crystalline phases, dielectric constant

## Abstract

Modern material science often makes use of polyvinylidene fluoride thin films because of various properties, like a high thermal and chemical stability, or a ferroelectric, pyroelectric and piezoelectric activity. Fibers of this polymer material are, on the other hand, much less explored due to various issues presented by the fibrous form. By introducing carbon nanotubes via electrospinning, it is possible to affect the chemical and electrical properties of the resulting composite. In the case of this paper, the focus was on the further improvement of interesting polyvinylidene fluoride properties by incorporating carbon nanotubes, such as changing the concentration of crystalline phases and the resulting increase of the dielectric constant and conductivity. These changes in properties have been explored by several methods that focused on a structural, chemical and electrical point of view. The resulting obtained data have been documented to create a basis for further research and to increase the overall understanding of the properties and usability of polyvinylidene fluoride fiber composites.

## 1. Introduction

Polymer materials are an ever-expanding and always attractive topic for a number of scientific fields, such as material engineering, electro-technology or even biomedical purposes. In particular, fluoropolymers have gained prominence in recent years [1,2,3,4,5]. Their excellent biocompatibility and high resistance to chemical and physical stress make them useful and sought after, but at the same time cause their patterning to be difficult [6]. The most active and broadly successful of these fluoropolymers is polyvinylidene fluoride (PVDF). Even though PVDF requires a specific approach to patterns, for example sputtering, otherwise widely used methods for patterning other polymers cannot be used, and a sufficient and reliable patterning can today be achieved by a number of methods, most commonly spin-coating of PVDF solution in a polar solvent [7,8]. In the case of nanofiber production, electrospinning arose as the method with the best results and most control over the parameters of the resulting fiber, such as the diameter, inclusions and to some extent even crystalline phases [9,10]. For this reason, electrospinning was also used to create the materials described in this paper.

**Figure 1:**
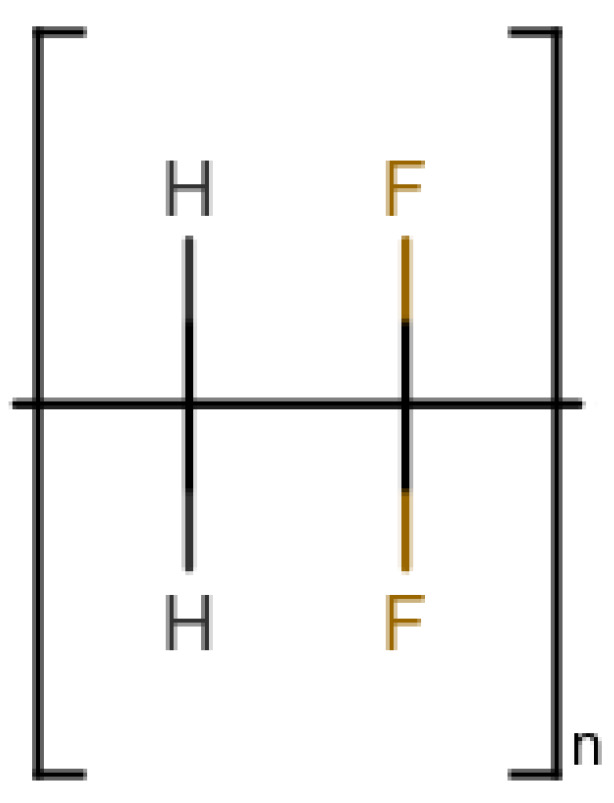
Poly(1,1-difluoretylene)–Vinylidene fluoride polymer.

PVDF has a number of desirable properties for many applications. It is a polymer with a high degree of thermoplasticity and low reactivity, and because of that it is used in many fields, from semiconductors via chemistry to biology. The creation of PVDF is achieved by the polymerization of vinylidene difluoride into a polymer chain (Figure 1).

PVDF can show different properties depending on the conformation of the polymer chain and production parameters. It is possible to achieve the poling of the molecular chain under tension by mechanical stretching, giving it ferroelectric, pyroelectric and piezoelectric properties [11,12,13,14,15]. In recent years, PVDF materials were subjected to doping by several interesting materials, namely BiFeO_3_, TiO_2_ and carbon nanofibers, modifying and enhancing its properties.

As with other polymeric structures, efforts to enrich PVDF with other materials have been explored as well. PVDF films and composite polymers have been blended with carbon nanotubes (CNTs) [16,17,18], and studies have found this process to enhance the β-phase, as well as all the electric and chemical properties that go with it, even making them a viable candidate for energy storage uses [19]. Since CNTs are widely used today, mainly for their large surface areas rich with electrons but also for their flexibility and durability, their incorporation into PVDF was a logical next step. Their presence in the polymer improved the pyroelectric, ferroelectric and piezoelectric properties in a major way [20,21,22]. The possible options of this material blend also qualify it as a candidate for smart materials [9]. While these inclusions have already been performed and evaluated on PVDF in some of the papers referenced above, the polymer was mostly in the form of a thin film or in a bulkier state. The binding mechanism, however, remains largely unexplored. The novelty of this paper, then, is the exploration of the properties and evaluation of the results of PVDF fibers bonded with carbon nanotubes in a composite material produced by electrospinning.

## 2. Materials and Methods

The PVDF material used in the measurements described in this paper was in the form of fibers (Mw = 275,000 g/mol) manufactured by electrospinning from 15 wt% PVDF solution (Sigma Aldrich, Munich, Germany) in a solution blend of dimethyl sulfoxide and acetone (Penta, Prague, Czech Republic) in a volume ratio of 7/3. The resulting material created under a constant voltage of 50 kV took the form of a 25 µm thick fiber mat. The process of electrospinning was performed on 4-spin equipment (Contipro, Dolní Dobrouč, Czech Republic) at a feeding rate of 20 µL × min^−1^ through a thin needle with a diameter of 1.067 mm (17G). An aluminum foil-covered rotation collector (Contipro, Dolní Dobrouč, Czech Republic) was used to gather the resulting fibers at a speed of 2000 rpm for 30 min. The distance between the tip of the needle and the collector was kept constant at 20 cm. The resulting nonwoven mats were left to dry overnight at laboratory temperature. Fibers crated by this process were 195.2 nm thick.

The nanotubes used in the experiments in this paper are NANOCYL NC7000 thin multiwall carbon nanotubes (CNTs, Sigma Aldrich, Munich, Germany) with an average diameter of 9.5 nm, average length of 1.5 µm and 90% carbon purity. 1 wt% of CNTs was dispersed in the 15 wt% PVDF solution using an ultrasound probe (Bandelin, Berlin, Germany) and were further electro-spun under the same process conditions mentioned for the neat PVDF solution, except for the feeding rate. That was optimally set at 80 µL × min^−1^ due to the enhanced ability to withdraw a drop of CTNs/PVDF precursor during the electrospinning process.

Scanning electron microscopy images were obtained by using a high-resolution scanning electron microscope FEI Verios 460L (FEI, Brno, Czech Republic).

Raman spectra were taken by a WITec alpha300 R device (WITec, Ulm, Germany) at an excitation wavelength of 532 nm and power of the laser of 1 mW. The signal gained from this measurement was reconstructed from 50 accumulations under an integration time of 20 s.

Photoluminescence spectra were acquired on the same device as Raman spectroscopy, with a laser power of 4 mW at a 355 nm wavelength. Through a 40× objective, the results were accumulated 20 times over a 5 s integration.

The device used for the acquisition of XPS spectra was an AXIS Supra X-ray photoelectron spectrometer (Kratos Analytical, Manchester, UK). The resulting information were captured under an emission current of 15 mA and resolution of 20 for wide spectra and 80 for the element-specific spectra. The fitting of the spectra was done using CasaXPS software (Casa Software Ltd., Teignmouth, UK).

Data from FTIR (Bruker, Billerica, MA, USA) were acquired in transmission mode over 512 iterations with a resolution of 1 cm^−1^.

An XRD analysis was performed with the X-ray powder diffractometer Rigaku SmartLab 3 kW (Rigaku Corporation, Tokyo, Japan) in the Bragg-Brentano configuration. Diffraction patterns were obtained between 10° and 50° (2θ) with Cu Kα radiation.

The dielectric properties were measured by a Novocontrol Alpha Analyzer device (Novocontrol Technologies, Montabaur, Germany) in the frequency range of 1 to 100,000 Hz. All of the measurements mentioned in this chapter were carried out at room temperature.

## 3. Results and Discussion

**Figure 2:**
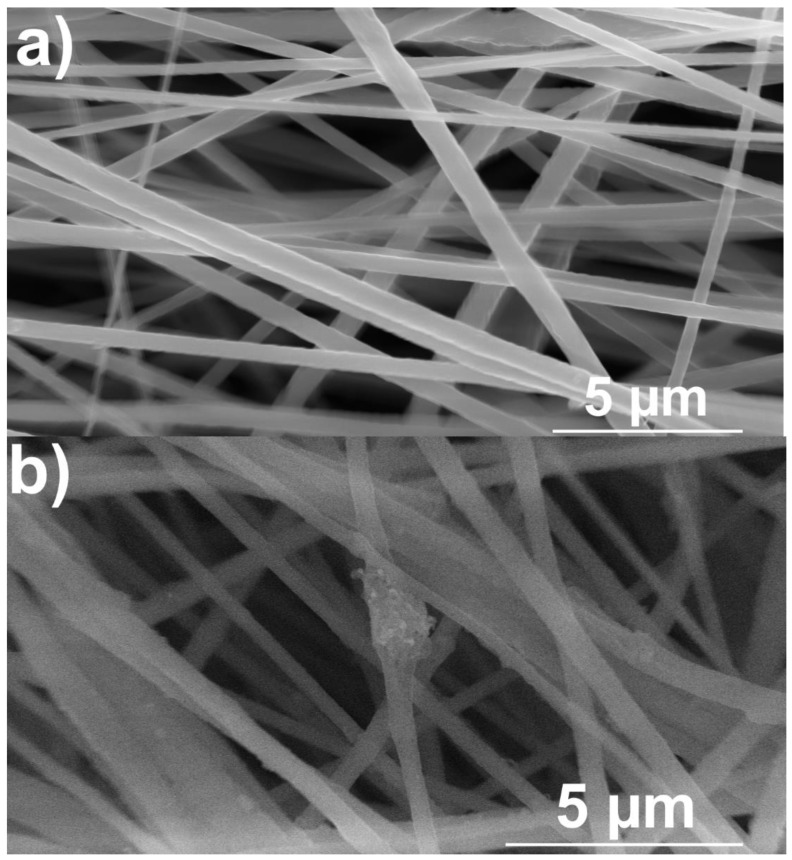
SEM images of pure PVDF fibers (**a**) and PVDF fibers with CNTs (**b**).

One of the more challenging issues is the dispersion of the carbon nanotubes within the PVDF solution before electrospinning and the distribution in the final product that it directly affects. For the illustration of surface changes, SEM images were taken of both the pure PVDF fibers and the PVDF fibers modified with CNTs (Figure 2). While the pure PVDF fibers have fairly clean and smooth surface (Figure 2a), a number of bumps and bulges has been detected in the combined material (Figure 2b). Not only are the CNTs incorporated inside the PVDF fibers, but we can see them protruding out in some places. The agglomeration in the center of the mixed material image (Figure 2b) also points to the possibility of the filler material forming clusters that are large enough to be clearly visible under scanning electron microscopy. The CNTs are, however, mostly incorporated into the fibers from the inside, and no separate formations of pure CNTs material without any attachment to the PVDF fibers have been detected.

**Figure 3:**
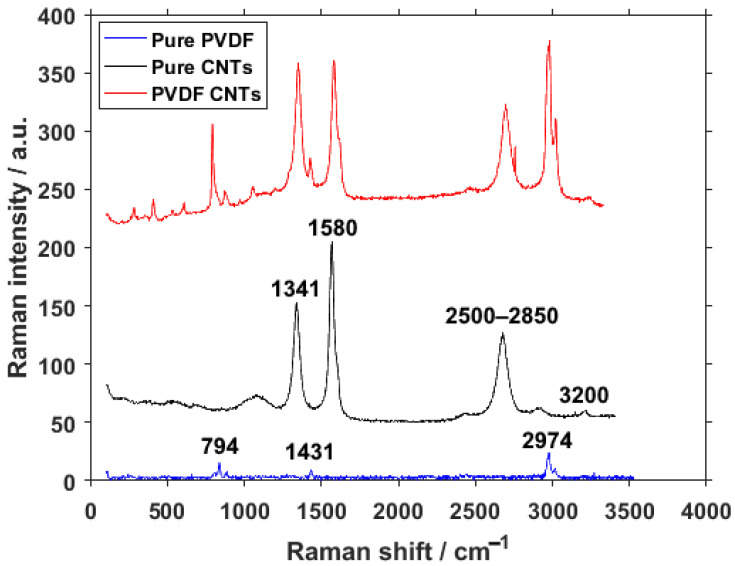
Raman spectra of pure PVDF fibers, pure CNTs and PVDF fibers with CNTs.

Samples of PVDF with carbon nanotubes were subjected to Raman spectroscopy. Figure 3 shows the wide Raman spectrum of the materials. The individual spectra are horizontally shifted for clarity. Carbon nanotubes have a number of highly visible bands present in the spectrum. The most prominent are D-band at around 1341 cm^−1^ caused by graphene structure disorder and, at 1580 cm^−1^, the G-band representing vibrations of the C–C bond [23]. Two smaller bands of note are located between 2500 and 2850 cm^−1^, assigned to the 2D group [24], and there is a very minor band around 3200 cm^−1^, representing a slightly displaced combined G + D’ band [25]. The pure CNTs part of the spectra offers one more piece of information about the material. The ratio of D- and G-bands can be used to determine the concentration of CNTs with different numbers of walls.

While the material is purchased and the documentation containing this information is available from the manufacturer [26], the spectrum lacks the presence of the radial breathing mode (RBM), which should be located between 150 and 200 cm^−1^. This absence points to a very low to no presence of single-wall carbon nanotubes (SWCNTs) [27]. For pure PVDF, the signal is very low, but there are still three visible bands. The band at around 794 cm^−1^ is assigned to the rocking motion of CH_2_ and is a typical band for α-phase rich PVDF. The band around 1431 cm^−1^ is caused by bending CH_2_ vibrations [28,29], present in all three crystalline phases of PVDF but mainly in the β- and γ-phases. The band at 2974 cm^−1^ is usually attributed to CH_2_ symmetric stretching [30], commonly associated with the β-phase.

In the spectrum of PVDF combined with CNTs, all the previously mentioned peaks are present and visible, but their intensities are put into relative perspective to each other. The change in ratio of the D/G bands can be ascribed to processes during the chemical bonding of the two materials, specifically the reduction of the crystallite size and an increased number of formed defects.

**Figure 4:**
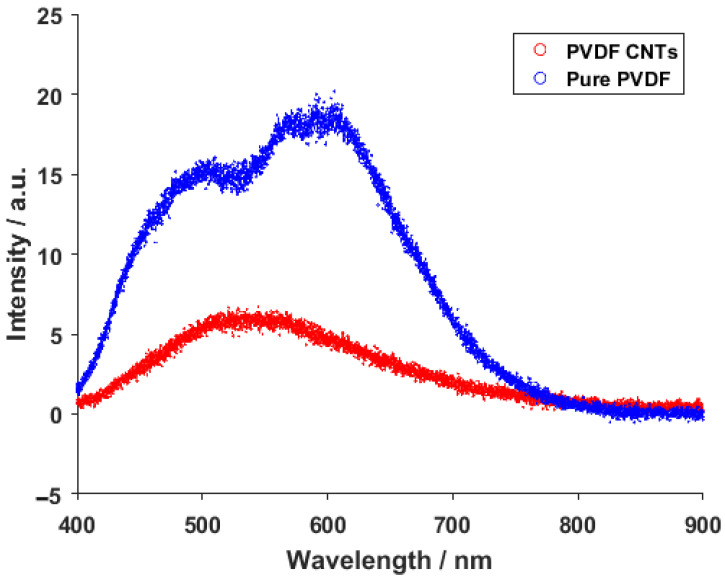
Photoluminescence spectra of pure PVDF fibers and PVDF fibers with CNTs.

The resulting emission spectra from the photoluminescence measurement of pure PVDF and PVDF with integrated carbon nanotubes can be seen in Figure 4. The recorded spectrum of pure PVDF shows two very visible peaks around 500 and 590 nm. These peaks can be assigned to ^4^F_9/2_—^6^H_15/2_ and ^4^F_9/2_—^6^H_13/2_ transitions, respectively [31,32]. This response was expected under the excitation wavelength of 354 nm. With the addition of carbon nanotubes, however, the previous signals of the transition got converted into a single wide peak at around 530 nm. This change can be attributed to bonds forming in the material after the introduction of the carbon nanotube, and not to the CNTs itself, since the standard photoluminescence peaks belonging to carbon nanotubes are usually located in higher wavelengths [33,34].

**Figure 5:**
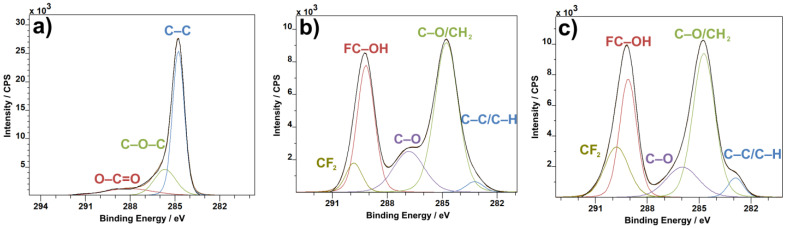
C1s XPS spectra of CNTs (**a)**, PVDF (**b**) and PVDF with CNTs (**c**).

The C1s XPS spectra of the analyzed material show a standard set of bands expected for carbon nanotubes [35] and PVDF [36]. The C–C band at 285 eV (Figure 5a) is one of the main pillars of any carbon XPS measurement and can be used as an identifier for the presence of graphite in almost any formation, like sheets, nanotubes or others. This peak is overshadowed by the C–O/CH_2_ band in the combined material (Figure 5c), as the material ratio of PVDF to CNTs is heavily in favor of PVDF. The combined C1 spectra also show a slight change in the ratio of the C–O/CH_2_ band to the FC–OH band, in favor of the former, when compared to pure PVDF (Figure 5b). The ratio of CF^2^ to the FC–OH bond increases in favor of CF^2^ in the combined material as well, representing the bonding process of CNTs to PVDF.

**Figure 6:**
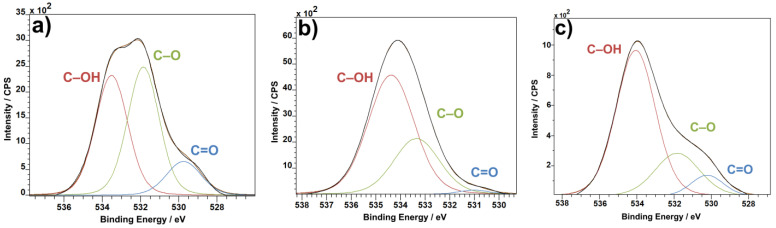
O1s XPS spectra: CNTs (**a**), PVDF (**b**) and PVDF with CNTs (**c**).

In the case of the O1s XPS spectra, the C–OH band from both CNTs (Figure 6a) and PVDF (Figure 6b) gets carried over to the combined material (Figure 6c), as expected. The most prominent band from pure CNTs, the C–O band, which is present in pure PVDF as well, is distinctly reduced in the combined material in favor of the C=O band, which increases in comparison to both pure CNT and pure PVDF. The O=C–O bond from the C1s spectra (Figure 5) and C=O from the O1s spectra (Figure 6) represent the ends of carbon nanotubes. When introduced to PVDF, the ends of CNTs bind to the polymer material, thus causing the C=O bond to decrease in concentration. This applies for the C–O bond as well, and, in turn, the C–OH bond, which is the bond of CNTs to PVDF, gains in prominence.

**Figure 7:**
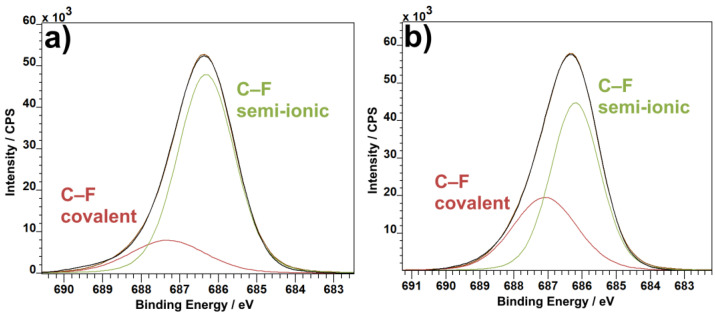
F1s XPS spectra: PVDF (**a**) and PVDF with CNTs (**b**).

The F1s spectra show a change in the ratio of covalent and semi-ionic bonds. Although the change is not enormous, semi-ionic bonds are more prevalent in pure PVDF material (Figure 7a) than in the combination of PVDF with CNTs (Figure 7b). The change in bond concentration reflects the shift of crystalline phases. Where semi-ionic bonds are more oriented than covalent bonds, the same can be said about the β and γ phases being more oriented than the α phase. The F1 spectra could then be read not only as a change in bonds in favor of covalent, less oriented bonds, but at the same time as an increase in concentration of the less oriented α phase.

**Figure 8:**
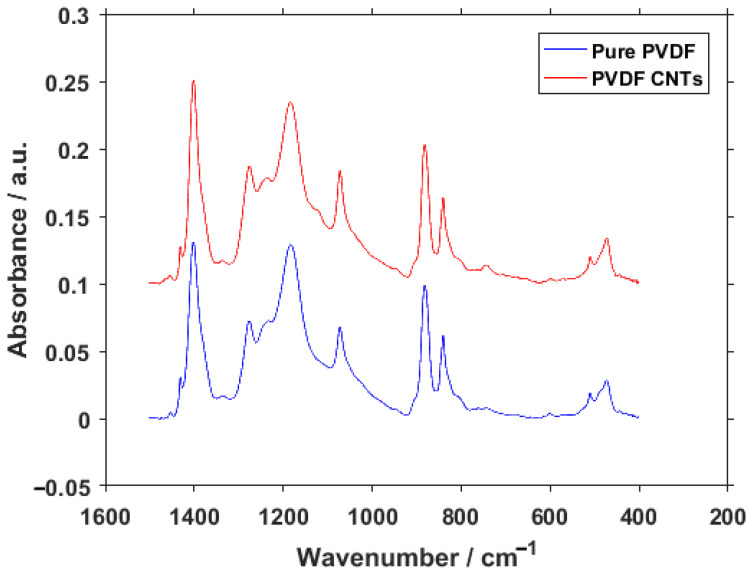
FTIR spectra of pure PVDF fibers and PVDF fibers with CNTs.

The FTIR measurement was performed in order to obtain the percentages of different PVDF phases with and without the enrichment by CNTs. The graphical interpretation of the result can be seen in Figure 8. At first glance, there are almost no differences between the spectra. The only visible variation is the presence of a peak on PVDF with CNTs at 743 cm^−1^, whereas this location is much flatter in pure PVDF. This location belongs to the α-phase of PVDF. Together with the peak at 840 cm^−1^ belonging to the β-phase, they can tell us the ratio of these two phases present in the material [37]. To fully understand what this ratio means, the relative concentration of phases was calculated from the results. The relative fraction of phases for pure PVDF was 13.45% for α, 82.52% for β and 4.03% for γ. This was changed with the addition of CNTs to 17.80% for the α-phase, 74.37% for β and 7.83% for γ. From these results, it could be seen that the inclusion of CNTs in PVDF fibers caused an increase mainly in the α-phase but also in the γ-phase of PVDF at the expense of the β-phase. This result supports the conclusions gained from XPS, namely the F1 spectra (Figure 7), which suggested a shift in concentration towards the α-phase based on the increase of covalent bond representation. Changes in the concentration suggest that the fibers bond with the PVDF structure and alter the polymer chain orientation, rather than just being lodged in between the individual fibers. It is also interesting to note that the previous measurements with carbon flakes incorporated into the PVDF fibers caused a decrease in α-phase concentration [38], as opposed to the CNTs presented in this paper.

**Figure 9:**
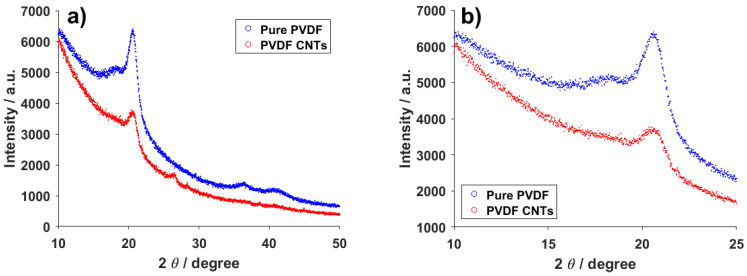
XRD (**a**) wide and (**b**) focus spectra of pure PVDF fibers and PVDF fibers with CNTs.

The XRD measurement revealed three places of interest in the spectra of pure PVDF and CNTs-infused PVDF. The first important peak is located at 27° (Figure 9a) and can only be seen in the spectrum belonging to PVDF with CNTs. Since this peak belongs to carbon, its presence in the combined material and absence in the pure PVDF spectrum is to be expected. Previous measurements on this material with powdered carbon instead of CNTs showed this peak to be much more pronounced [38], so the conformation of carbon plays a role when determining the intensity of the carbon response from this method. The other two places of interest belong to the α-phase of PVDF and the combination of the α and β-phases at around 18° and 21°, respectively (Figure 9b) [39]. In the combined material, it seems as if the α-phase peak has almost disappeared, though it can still be seen. This is caused by the change in ratio of the first and second peak. For the pure PVDF material, the ratio of the 18° and 21° peaks is clearly different than in the combined material. Specifically, with the introduction of CNTs into PVDF the ratio shifts more toward the 18° peak, representing a higher concentration of the α-phase than in the pure material. This corresponds with the results from other measurements described in this paper.

**Figure 10:**
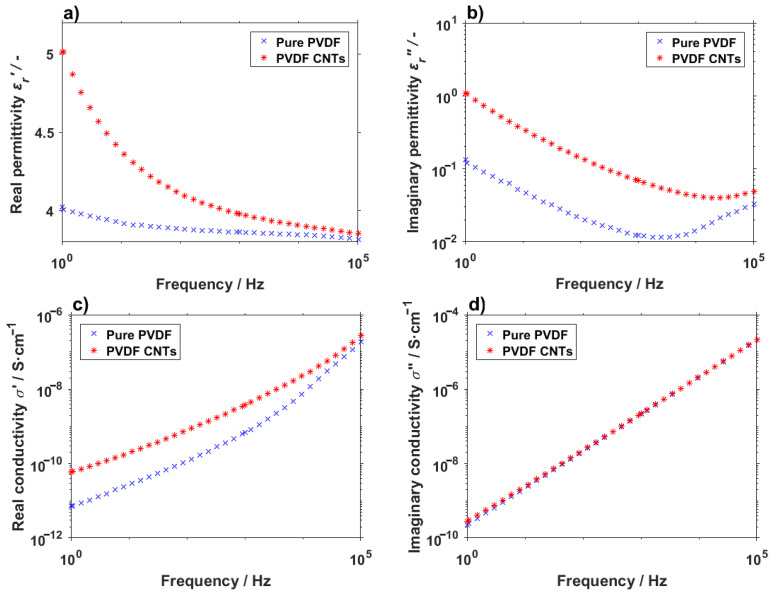
(**a**) Real and (**b**) imaginary permittivity and (**c**) real and (**d**) imaginary conductivity of pure PVDF fibers and PVDF with carbon nanotubes.

Carbon nanotubes are also often explored and exploited because of their electrical properties [40]. The addition of CNTs into PVDF was expected to have an effect on the overall electrical behavior of the material. From the dielectric measurements depicted in Figure 10 it appears that the effect of the carbon nanotubes presence cannot be simply disregarded. On lower frequencies, the dielectric constant (*ε_r_′*) is noticeably higher, even though the difference decreases rapidly with an increasing frequency. The imaginary part (*ε_r_″*), a.k.a. dielectric loss, follows a similar trend for both the pure and doped PVDF material, but with different starting values at the lower frequencies and almost blending together on higher frequencies. More interesting information can be obtained from the conductivity part of Figure 10. As expected, the introduction of the carbon nanotubes increases the conductivity of the fibers, though the effect is mainly noticeable at lower frequencies, as was the case for the dielectric constant. In the case of the imaginary part of the conductivity (*σ″*), the values for the pure and doped PVDF are virtually indistinguishable. These trends are all in line with the expectations and measurements made on PVDF films, where the electrical properties should not be too different [41,42].

## 4. Conclusions

During the electrospinning process used to include carbon nanotubes into polyvinylidene fluoride fibers upon creation, the CNTs formed chemical bonds with PVDF and were not just mechanically inserted into a previously existing material. The resulting bonds were detectable by XPS and photoluminescence. Such an interaction affected the properties and structure of the polymer, most noticeably in the ratio of crystalline phases, as presented by Raman spectroscopy, FTIR and XRD. The concentration of the α-, β- and γ-phases, not to mention the presence of the nanotubes on their own, also had an effect on the permittivity and conductivity of the material and could allow the resulting fibers to be modified for specific use on demand. The specific acquired results are valid for the 1 wt% of CNTs in the 15 wt% solution used for electrospinning. The performed inclusion and analysis of CNTs into PVDF fibers has not been described before, and it offers an important look into the mechanisms of fiber-inclusion interactions, opening new possibilities for the utilization of polyvinylidene fluoride fibers with the inclusions of other materials.

## Data Availability

The authors declare that, to the best of their knowledge, all data and material comply with field standards. Data are available by request.
